# Engineering of microbial cells for L-valine production: challenges and opportunities

**DOI:** 10.1186/s12934-021-01665-5

**Published:** 2021-08-30

**Authors:** Hui Gao, Philibert Tuyishime, Xian Zhang, Taowei Yang, Meijuan Xu, Zhiming Rao

**Affiliations:** The Key Laboratory of Industrial Biotechnology, Ministry of Education, School of Biotechnology, Jiangnan University, Wuxi, 214122 Jiangsu China

**Keywords:** L-Valine biosynthesis, Feedstocks, Microbial cell factories, Metabolic engineering, Fermentation

## Abstract

L-valine is an essential amino acid that has wide and expanding applications with a suspected growing market demand. Its applicability ranges from animal feed additive, ingredient in cosmetic and special nutrients in pharmaceutical and agriculture fields. Currently, fermentation with the aid of model organisms, is a major method for the production of L-valine. However, achieving the optimal production has often been limited because of the metabolic imbalance in recombinant strains. In this review, the constrains in L-valine biosynthesis are discussed first. Then, we summarize the current advances in engineering of microbial cell factories that have been developed to address and overcome major challenges in the L-valine production process. Future prospects for enhancing the current L-valine production strategies are also discussed.

## Background

Branched-chain amino acids (BCAAs) comprising valine, isoleucine and leucine, are essential amino acids that are key components of human and animal nutrition, and need to be obtained from the daily diet. [1, 2]. Besides to their important role as build blocks of proteins, BCAAs earned the greatest reputation to be applied in a large number of industrial applications including the food, feed, cosmetic and pharmaceutical industry. Notably, L-valine is the α-amino acid with chemical formula C_5_H_11_NO_2_ that is commercially important for the need to supplement food or feed and to use in medical treatment and biochemical synthesis precursors [3]. L-valine can improve the lactation function of breeding animals and has been considered as one of the limiting amino acids in animal feed for poultry and pigs [4–6]. The addition of L-valine can make cosmetics have moisturizing function and promote the synthesis of collagen. In the pharmaceutical industry, L-valine is widely used as a component of third-generation amino acids infusion and is highly tolerant to the synthesis and decomposition of muscle protein; it has a critical role in pharmacological nutrients for patients with chronic liver disease [7]. Moreover, L-valine activate the PI3K/Akt1 signaling pathway and inhibit the activity of arginase to increase the expression of NO. Therefore, L-valine can enhance phagocytosis of macrophages to drug-resistant pathogens and serves as a chemical building block for anti-viral drugs and antibiotics (monensin, cervimycin and valanimycin) [8]. Because of the increased world amino acids needs, it is estimated that the production of amino acids can yield revenue of US$ 25.6 billion by 2022 and animal feed amino acids (BCAAs) is the largest and fastest-growing market with expected revenue of US$10.4 billion by 2022). In recent years, the global L-valine market has been growing rapidly with a compound annual growth rate (CAGR) of 65% from 2016 to 2019, and is expected to maintain a CAGR of 24% from 2020 to 2023, among which feed grade L-valine has the most significant growth rate, with a CAGR of 48% from 2015 to 2020 [9]. Taking into consideration, this may change the near future research for efficient production of animal feed amino acids. Thus, there is a strong impetus for improving L-valine production. Traditionally, L-valine production is centered on the hydrolysis process with subcritical water technology which use cheap feedstock solid waste (mainly including skin, feathers, viscera, blood, bones and residual meat produced in poultry processing) consisting of proteins which composed of a variety of amino acids [10]. Chemical synthesis or enzymatic processes have been also applied for L-valine production [11]. However, the complexity of these process and suboptimal production prompt the above strategies less attractive and nonsustainable. The direct fermentation of hexoses (mainly glucose) has therefore become one of the great promising process for the industrial-scale production of L-valine. Fermentation pathway with the aid of microorganisms has received increasing attention because of the new genetic engineering tools applied to maximize the yield, titer and productivity [12, 13]. Moreover, a great deal of effort has been devoted to correctly directing metabolic flux to the L-valine pathway through overexpression of the rate-limiting enzymes, transporters, and deactivation of the competitive pathways. L-valine-producing strains mainly includes *Corynebacterium glutamicum subsp. flavum* [14, 15], *Bacillus subtilis* [16], *Bacillus licheniformis* [17, 18], *Saccharomyces cerevisiae* [19], *Escherichia coli* [20–22], and *Corynebacterium glutamicum* [23]. However, it is still difficult to achieve high production of L-valine because of the interdependency between pathways in engineered strains. The optimization of L-valine biosynthetic pathways in a single host is also hampered by unexpected metabolic burdens that may trigger low energy and leave the host in an unstable physiological state [24, 25]. L-valine of the biosynthetic pathway in a single host body is hampered by unexpected metabolic burden which could cause low energy, make host in unstable state of physiology. In this manuscript, recent engineering strategies for the production of L-valine by microbial cell factories are reviewed with accompanying examples. We also suggest the potential approaches for optimizing L-valine biosynthesis pathway with the help of system and synthetic biology.

## Pathway of L-valine biosynthesis in *C. glutamicum*, *E. coli* and *B. subtilis*

**Fig. 1:**
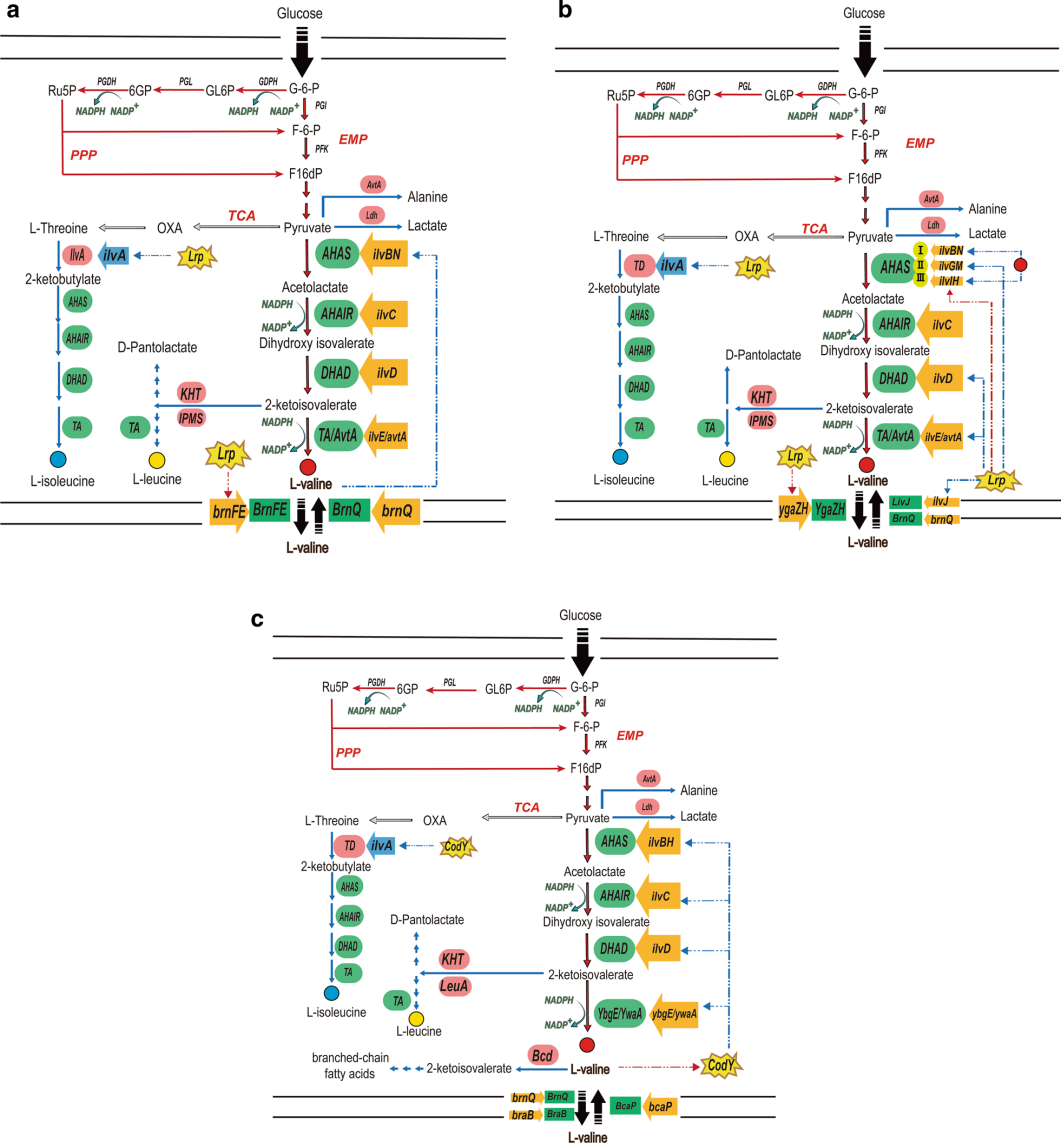
Overview of biosynthetic pathway of L-valine in *C.glutamicum, E. coli and B. subtilis.* The solid red arrow shows the L-valine synthesis pathway and the solid blue arrow shows the inhibition pathway of L-valine synthesis. The red dotted line shows activation and the blue dotted line shows inhibition. **a** overview of biosynthetic pathway of L-valine in *C.glutamicum*; **b** overview of biosynthetic pathway of L-valine in *E. coli*; **c** overview of biosynthetic pathway of L-valine in *B. subtilis*. AHAS, acetohydroxyacid synthase; AHAIR, acetohydroxyacid isomeroreductase; DHAD, dihydroxyacid dehydratase; TA, transaminase B; AvtA, alanine transaminase; GPDH, glucose-6P dehydrogenase; PGL, 6-phosphogluconolactonase; PGDH, 6P-gluconate dehydrogenase; PGI, phosphoglucose isomerase; PFK, phosphofructokinase; Ldh, Lactate dehydrogenase; IlvA, L-threonine dehydratase; KHT, ketopantoate hydroxymethyltransferase; IPMS, 2-isopropylmalate synthase; LeuA, 2-isopropylmalate synthase; G-6-P, glucose 6-phosphate; F-6-P, fructose 6 phosphate; F16dP, Fructose 1,6 diphosphate; OXA, oxaloacetate; GL6P, gluconolactone 6-phosphate; 6PG, 6-phospho gluconate; Ru5P, ribulose 5-phosphate; EMP, Embden-Meyerhof-Parnas; TCA, tricarboxylic acid cycle; PPP, pentose phosphate pathway

The synthesis of L-valine starts from pyruvate and involves four enzymes, namely acetohydroxyacid synthase (AHAS), acetohydroxyacid isomeroreductase (AHAIR), dihydroxyacid dehydratase (DHAD), and transaminase B (TA) [26]. The synthesis and regulation of L-valine in *C. glutamicum, E. coli and B. subtilis* are not identical (Fig. 1a,b,c). In *C. glutamicum*, AHAS is the initiation enzyme of three branched chain amino acids and a key enzyme in the synthesis pathway of L-valine. Encoded by *ilvB* and *ilvN*, AHAS catalyzed two molecules of pyruvate to form 2-acetolactate (Fig. 1a). This enzyme consists of two large subunits and two small subunits to form the catalytic domain and structural domain. Environmental oxidation level regulates the activity of AHAS, and cofactors flavin adenine dinucleotide (FAD) and Mg^2+^ are also required for AHAS activity. AHAIR encoded by *ilvC* catalyzes the isomerization reduction of 2-acetolactate to form dihydroxyisovalerate, whose activity requires Mg^2+^ and NADPH [23]. DHAD ecoded by *ilvD* catalyzed the dehydration of dihydroxyisovalerate to 2-ketoisovalerate. Finally, TA encoded by *ilvE* converts 2-ketoisovalerate to L-valine and this reaction also catalyzed by AvtA (encoded by *avtA*) in *C. glutamicum*. The transcription of *ilvB*, *ilvN* and *ilvC* genes is regulated by the operon *ilvBNC*, which has three different promoters that respectively start the transcription of *ilvB*, *ilvN* and *ilvC* genes to form three mRNA of different lengths. Because *ilvC* is located at the end of the operon *ilvBNC*, the transcription of *ilvC* gene was three times that of the other two genes, and the expression efficiency of *ilvC* gene was the highest among the three genes [26]. BrnFE is a two-component osmotic enzyme in *C. glutamicum*, which is responsible for the export of L-valine. The expression of BrnFE is regulated by the global regulatory factor Lrp. Lrp is activated to promote the expression of *brnFE*. The protein BrnFE transports L-valine to the outside of the cell and the import of L-valine is performed by the protein BrnQ.

The regulation of L-valine synthesis in *E. coli* appears to be more complex than in *C. glutamicum*. In contrast to *C. glutamicum*, there are three different AHAS isoenzymes in *E. coli* with different biochemical and regulatory properties (Fig. 1b). AHAS I is encoded by *ilvBN*, AHAS II is encoded by *ilvIH*, and AHAS III (encoded by *ilvIH*) is highly similar to *C. glutamicum*. 172 amino acids in *C. glutamicum* (encoded by *ilvN*) have 39% homology with 163 amino acids in *E. coli* (encoded by *ilvH*). It contains an N-terminal ACT domain [26]. The sites responsible for L-valine feedback inhibition are located in the small subunits of AHAS I (encoded by *ilvN*) and AHAS III (encoded by *ilvH*), while AHAS II (encoded by *ilvGM*) is resistant to L-valine. At the transcriptional level, the attenuation of *ilvGMEDA *(*ilvE* encodes transaminase, *ilvD* encodes dihydroxyacid dehydratase, *ilvA* encodes L-threonine dehydratase) operon is mediated by three BCAAs (L-valine, L-leucine, L-isoleucine), and the absence of L-valine and L-leucine will trigger the attenuation control of *ilvBN* operon. Similar to BrnFE in *C.glutamicum*, YgaZH in *E. coli* is responsible for exporting L-valine, L-leucine and L-isoleucine. YgaZH is activated by the transcription of global regulatory factor Lrp, which is also involved in the synthesis of L-valine. It activates the expression of AHAS III (ecoded by *ilvIH*) isozyme, while L-leucine inhibits *ilvIH* operon expression by blocking Lrp binding. Lrp also inhibits *ilvGMEDA* operon expression. In *E. coli*, BCAAs is imported through BrnQ and LivJ, and Lrp also inhibits the transport of LivJ protein.

In *B. subtilis*, as in *E. coli* and *C. glutamicum*, L-valine synthesis begins with pyruvate and proceeds through intermediates acetolactate, dihydroxyisovalerate, 2-ketoisovalerate, and finally L-valine. The enzymes involved in this pathway are AHAS or ALS (encoded by *ilvHB* or *alsS*, respectively), AHAIR(encoded by *ilvC*), DHAD (encoded by *ilvD*), YbgE or YwaA (encoded by *ybgE* and *ywaA*, respectively) [16] (Fig. 1c). AHAS is involved in the synthesis of L-valine as well as leucine and isoleucine, while ALS is specific in the synthesis of L-valine and does not participate in the synthesis of other branch amino acids. YbgE and YwaA belong to transaminases, but the transaminase activity of YbgE is much higher than that of YwaA [27]. Similar to Lrp in the *E. coli*, CodY in *B. subtilis* is also a global regulatory factor, which exists in gram-positive bacteria with low G + C (G + C base pair content < 50%), such as *B. subtilis*, *Clostridium acetobutylicum*, and *Staphylococcus aureus *[28]. Branched chain amino acids can activate CodY. When L-valine is abundant in cells, CodY will inhibit *ilvBHC*, *ilvA*, *ilvD*, ygaE, ywaA. L-valine is decomposed to 2-ketoisovalerate through the enzyme Bcd which is also known as leucine dehydrogenase (LeuDH). This enzyme can be found in *Bacillus* and the thermophilic *Clostridium*. Bcd can naturally and reversely catalyze the deamination of branched-chain amino acids to ketoanalogs [29]. BrnQ, BcaP and BraB (encoded by *brnQ*, *bcaP* and *braB* respectively) are three kinds of L-valine permeases in *B. subtilis*.[30].

## Pathway constraints and metabolic engineering strategies for L-valine production

**Fig. 2:**
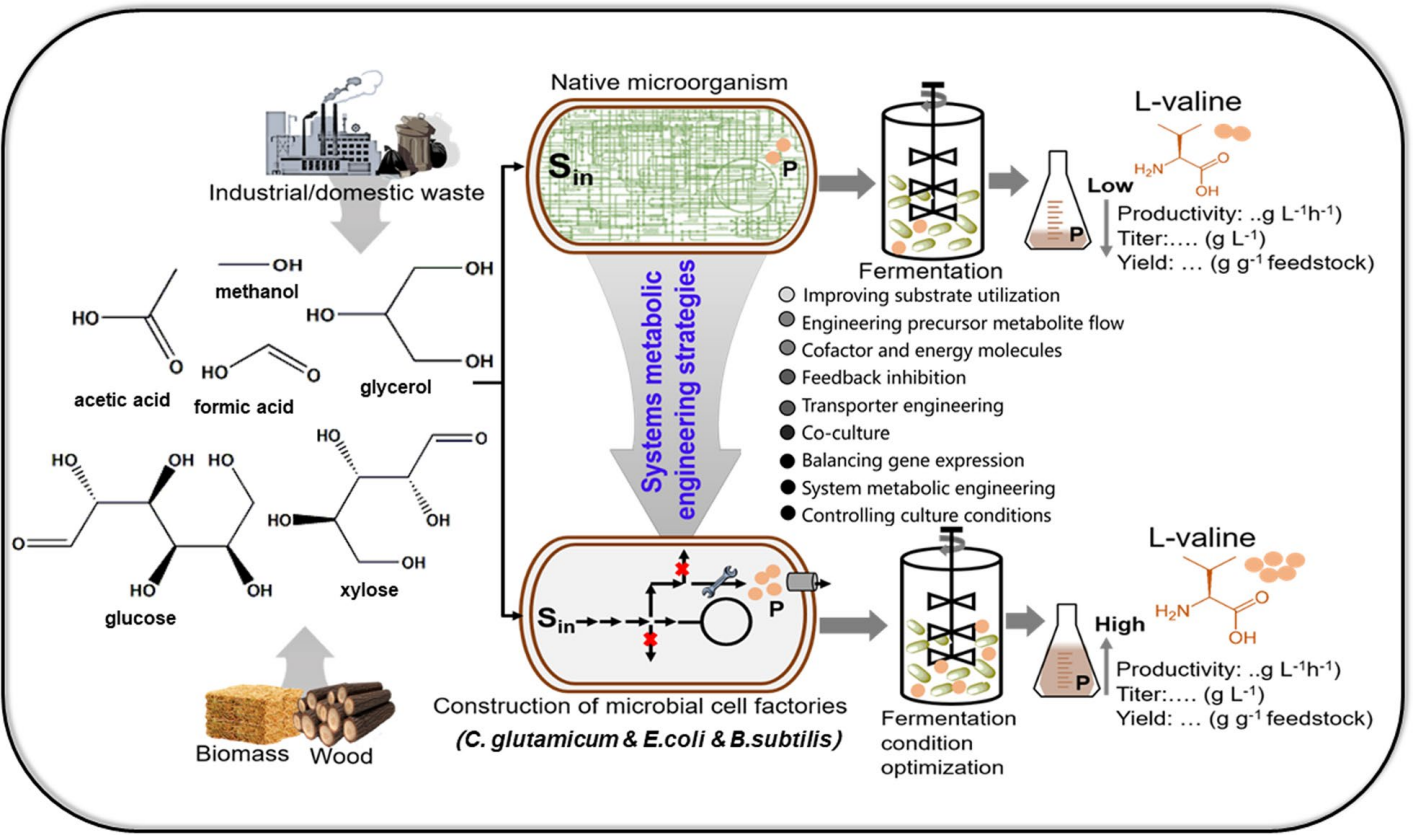
Engineering strategies for enhancing L-valine production in model organisms as microbial cell factories. L-valine is an essential branched chain amino acid that is widely used in industrial application. Most metabolically engineered strains prefer to use first generation feedstock such carbohydrate (glucose) as sole carbon and energy source. The choice of renewable carbon sources is also expanding and possibly, lignocellulosic raw material such as xylose and other carbon source; glycerol can be used for improved L-valine production. With the advancement of engineering, it is possible to streamline platform microorganisms such as *C. glutamicum*, *E. coli* and *B. subtilis* for better performance in terms of titer, productivity and yield

Microbial cell factories such as *C. glutamicum*, *E. coli*, *B. subtilis* and yeast strains are metabolically engineered to produce a broad variety of high value chemicals of everyday use [31]. Despite the effort to engineer industrial microorganisms for the production of L-valine, it encounters different metabolic bottlenecks in improving host cell physiology for high titer of L-valine with high yield and productivity. The first problem is that L-valine overproducing strains have heavily relied on first-generation feedstock, pure carbohydrates (e.g. glucose) as the sole carbon and energy source under monoculture regime [23, 26]. Second, the limiting step is that L-valine derives from build block pyruvate which is a central intermediate that contributes to other biochemical reactions. Third, the key metabolic enzymes and transports necessitated for L-valine biosynthesis in model strains, *C. glutamicum*, *E. coli* and *B. subtilis*, also function as prime in the biosynthesis of other BCAAs [23, 26]. Briefly, L-valine synthesis from pyruvate consists of four enzymes: AHAS, AHAIR, DHAD and TA. The key enzyme AHAS catalyzes either the condensation of two pyruvates to yield 2-acetolactate, leading to biosynthesis of L-valine and L-leucine, or condensation of pyruvate and 2-ketoisobyturate to form 2-aceto-2-hydroxybutyrate, leading to L-isoleucine biosynthesis [13, 32]. In addition, the last intermediate of L-valine synthesis, 2-ketoisovalerate is also the precursor for L-leucine and D-pantothenate biosynthesis. Fourth, the BCAAs exert the feedback inhibition on key enzyme AHAS (encoded by *ilvBN*) in *C. glutamicum*, and isoenzyme AHAS I and AHAS III (encoded by *ilvBN* and *ilvIH* respectively) in *E. coli* [32, 33]. In contrast, isoenzyme AHAS II (encoded by *ilvGM*) in *E. coli*, is insensitive to L-valine, but it is not expressed because of the frameshift mutation in *ilvG*. On the other hand, the AHAS II and AHAS III possess high affinity for 2-ketobutyrate than pyruvate, thus leading to L-isoleucine biosynthesis rather than L-valine biosynthesis [20]. To cope with this complexity and to unlock the full potential of microbial factories for L-valine production on an industrial scale, it is therefore advantageous to (i) improve substrate utilization (ii) improve precursor availability (iii) relieve negative regulatory circuits and enhance transport engineering (iv) balance genes expression. In recent research, several possible novel strategies have been proposed to improve the production of L-valine, such as taking advantages of abundant and cheap carbon sources, systematic metabolic engineering and culture conditions in fermentation (Fig. 2).

## Improving substrate utilization and expanding substrate spectrum

In response to the titer, yield and productivity necessitated, the choice of carbon substrate is the first crucial step for the success of bioprocess. Hence, substrate cost and availability are critical in industrial biomanufacturing [34]. Possible carbon sources for L-valine-producing bacteria include carbohydrates (glucose, glycerin, fructose, molasses, etc.) and organic acids (pyruvate, acetic acid, lactic acid, etc.). Ethanol and organic hydrocarbons can also be used as carbon sources. Currently, under monoculture conditions, overproducing strains of L-valine rely heavily on first-generation feedstock, pure carbohydrates (such as glucose) as the sole carbon and energy source. Glucose is a preferred carbon source of *C. glutamicum* and *E. coli*, its metabolism has been noted to exclusively depend on the glucose phosphotransferase system (PTS_glu_). In *C. glutamicum*, the uptake of glucose is catalyzed by a phosphotransferase system (PTS) consisting of EI and HPr (encoded by *ptsI* and *ptsH*, respectively), and glucose-specific EII permease EIIGlc (encoded by *ptsG*) [35]. The intracellular glucose is further phosphorylated using ATP and/or phosphate-dependent glucokinase [36]. To improve the efficiency of sugar utilization based on L-valine production, two traditional strategies; random mutation and fermentation optimization have been used and continued to be used for this purpose. The random mutagenesis is induced by chemical or physical mutagenesis to mutate the gene of the strain, and then the strain with excellent performance can be obtained through screening. By using multiple rounds of random mutagenesis and optimized batch fermentation, the mutant *C. glutamicum* strain VWB-1 exhibited a much higher consumption rate of glucose and high L-valine titer 29.85 g L^−1^ in comparison with the parent strain [37]. In addition, applying rational metabolic engineering offers a more advantageous method to improve the utilization of carbon source for L-valine production. In a study by Vogt and colleagues, deactivating the PTS-independent inositol transporter IolT1 which catalyzes glucose uptake, resulted in an increased glucose uptake rate by 43% and L-valine titer to 20 mM [38]. Considering that phosphoenolpyruvate (PEP) is required for PTS and it is also an important intermediate for L-valine biosynthesis, replacement of PTS with non-PTS could save more PEP for pyruvate availability as the main precursor to the desired product. For co-substrate utilization, increased glucose uptake was observed during glucose–maltose co-metabolism in pyruvate dehydrogenase complex-deficient *C. glutamicum*. The transcriptional regulator SugR inhibits the expression of genes *ptsI*, *ptsH* and *ptsG*. Thus, the addition of maltose increases the *ptsG* expression by counteracting the SugR-mediated repression, thereby co-substrate utilization improved L-valine production (Table 1) [39]. L-serine biosynthesis is mainly through the Embden–Meyerhof–Parnas (EMP) pathway. Co-substrate utilization by adding glucose and fructose as carbon sources for fermentation leads to higher cell growth and can produce more precursors to produce L-serine [40]. Possibly, the co-substrate utilization efficiency can be further applied to improve L-valine production. Metabolic engineering has been successfully used to extend the carbon substrate spectrum of microbial cell factories. Most metabolic engineering projects focus on de novo synthesis of target biological products, and renewable carbon sources can usually be obtained at a lower price. At present, new strategies for achieving sustainability and added value tend to use carbon feedstock from industrial waste streams. The composition of industrial waste is different from that of traditional raw materials, so people pay more attention to this alternative carbon source, such as pentose, xylose, arabinose, polyol glycerol and organic acid lactic acid [41]. Meanwhile, hemi-cellulosic pentose sugar (xylose) and glycerol-based production of valuable chemicals in *C. glutamicum* and *E. coli* have been described [9, 41]. Nevertheless, although these microbial cells have the ability to utilize different carbon resources, they have never previously used renewable feedstocks for L-valine production. Considering the challenges of global resources and food shortage, microbial cells that efficiently utilize the flexible feedstocks for amino acid production are highly desired.

**Table 1:** Major strategies and advancements in achieving and enhancing L-valine production

Host	Main engineering strategy	Culture condition	Performance			References
			Titer (g L^−1^)	Yield (mol mol^−1^ glucose)	Productivity (g L^−1^ h^−1^)	
*C. glutamicum* ATCC 13032	Alanine transaminase deactivated, AHAIS, AHAS and TA overexpressed	3.5 L Fed-batch fermentation 12.5% glucose, 31 °C, 72 h	31.1	0.28	0.43	[11]
*C. glutamicum* ATCC 13032	Increasing the expression of ptsG	50 mL shake flask fermentation, 4% glucose, 0.5% maltose, 2% acetate, 48 h	11.94	0.21	0.27	[39]
*C. glutamicum* ATCC 13032	L-threonine dehydratase and ketopantoate hydroxymethyltransferase deactivated. AHAIR, AHAS and DHAD overexpressed	100 mL flask batch cultivation, 4% glucose, 30 °C 48 h	10.7	ND	0.22	[50]
*C. glutamicum R*	Lactate dehydrogenase, alanine transaminase, CoA transferase, phosphotransacetylase, phosphoenolpyruvate carboxylase and acetate kinase deactivated. Variant of AHAIS and AHAS expressed	50 mL (flask) Fed-batch fermentation, 4% glucose, 33 °C, 24 h (anaerobic condition)	150	0.88	6.25	[66]
*C. glutamicum* ATCC 13869	L-threonine dehydratase, pyruvate dehydrogenase complex and alanine transaminase deactivated. AHAIR, AHAS and the genes *lrp1*/*brnFE* overexpressed	2 L Fed-batch fermentation, 12% glucose, 30 °C, 96 h	51	0.47	0.533	[68]
*C. glutamicum R*	Leucine dehydrogenase expression Feedback-resistant muntant AHAS	50 mL (flask) Fed-batch fermentation, 4% glucose, 33 °C, 24 and 48 h (anaerobic condition)	172.2 and 227	0.63	4.09 and 4.72	[65]
*C. glutamicum* ATCC 13032	Deactivating phosphoenolpyruvate carboxylase and quinone oxidoreductase combined with ALE AHAIS, AHAS, DHAD and TA overexpressed	50 mL flask batch cultivation, 4% glucose, 30 °C, 72 h	8.9	0.22	0.012	[22]
*C. glutamicum* VWB-1	Applying multiple random mutagenesis	5 L Fed-batch fermentation, 12% glucose, 31.5 °C 72 h	29.8	ND	0.41	[41]
*C. glutamicum* ATCC 13032	Random mutagenesis and selection Transcriptomic and metabolomics analysis	5 L Fed-batch fermentation, 5% glucose, 32 °C, 24 h	44.6	ND	ND	[46]
*Brevibacterium flavum JV16*	Alanine transaminase deactivated, AHAIS, AHAS and TA overexpressed	3.5 L Fed-batch fermentation, 2.5% glucose, 31 °C, 72 h	38.8	0.37	0.53	[11]
*Brevibacterium flavumMDV1*	Random mutagenesis by UV and Binary ethylenimine; BEI	12 L Fed-batch fermentation, 12.5% glucose, 30 °C, 64 h	68.8	ND	1.07	[12]
*E. coli* W	L-threonine dehydratase, Isopropyl malate synthase, ketopantoate hydroxymethyltransferase, malate dehydrogenase and pyruvate dehydrogenase phosphofructokinase deactivated (VAMF strain) AHAIS, AHAS, DHAD, TA and gene *lrp*/*ygaZH* overexpressed	100 mL Flask batch cultivation, 2% glucose, 0.3% sodium, 31 °C, 48 h	7.6	ND	0.15	[17]
*E. coli* W	AHAIS, AHAS variant, DHAD, TA and gene *lrp*/*ygaZH* overexpressed in VAMF strain	2 L Fed-batch fermentation, 2% glucose,, 31 °C, 55 h	32.2	ND	0.58	[18]
*E. coli* W	L-threonine dehydratase deactivated AHAIS, AHAS, DHAD, TA and gene *lrp*/*ygaZH* overexpressed	2 L Fed-batch fermentation, 2% glucose,, 31 °C 29.5 h	60.7	0.22	2.06	[19]
*E.coli* DH5α	Heterologous introduction of a feedback-resistant acetolactate acid synthase, exporter, redox cofactor balance, two-stage fed-batch fermentation	5 L Fed-batch fermentation, glucose concentration below 5 g/L, 37℃, 36 h	84	0.41	2.33	[57]
*Bacillus subtilis* 168	Pyruvate dehydrogenase complex, L-threonine dehydratase, isopropyl malate synthase and transcription factor σ (*sigF*) deactivated	20 mL Flask batch cultivation 3% sucrose, 1% mannitol, 37 °C 24 h	3.9	ND	0.19	[13]

*ND* not determined

The use of methane, methanol, formic acid and caron dioxide is increasing, although the formation of biological products through C1 carbon assimilation/utilization is currently less efficient than traditional carbon sources [34]. However, on the one hand, non-natural C1 model strains can achieve chemical production from carbon dioxide and reduce greenhouse gas emissions by using formic acid. On the other hand, methanol has a higher hydrogen/oxygen ratio than carbohydrates such as glucose, xylose, and glycerol. Methanol and formic acid can be ideal raw materials for the biosynthesis of organic compounds, alcohols and hydrocarbons [42]. The ability to convert captured carbon into value-added products attracts carbon capture and storage, sustainable chemical production and reduces our dependence on non-renewable fossil fuels. The successful design of fully autotrophic *E. coli* strains has opened the door for fast-growing model organisms to be used in carbon capture and the production of chemical substances and biofuels. Compared with existing microorganisms (such as algae and cyanobacteria), it has obvious advantages [43]. Therefore, the authors consider it is sought to produce L-valine from alternative raw materials that have no competition in the food and feed industries. Generally, the use of renewable raw materials reduces waste disposal costs and increases the economic value of the biological industry.

## Engineering precursor metabolite flow

In nature, microorganisms develop tight metabolic pathways, and carbon flux allocation at major nodes affects the yield of target products. Therefore, reducing the carbon flux to undesirable by-products through metabolic engineering strategies is a critical issue to achieve a high titer of a product with high yield and productivity [34, 44]. In order to maximize the metabolic flux of L-valine, it is important to ensure sufficient key precursors (pyruvate and 2-ketoisobutylate) through resolving rate-controlling steps and to enhance the expression of key enzymes in the biosynthetic pathway. Because pyruvate is an important intermediate in glycolysis, which serves as the precursor for several pathways within a biochemical network of an engineered host, it is worth noting that the biosynthesis pathways of BCAAs are partially overlapping and almost shared the same precursors (pyruvate and 2-ketoisovalerate) and enzymes (AHAS, AHAIR, DHAD, TA) [24, 26]. In consideration of this, most metabolic engineering strategies targeted the pyruvate dehydrogenase complex (PDHC) either directly or indirectly, overexpressing the L-valine production genes and suppressing the metabolic pathways of unwanted by-products. Different enzymes (Table 1) have been deactivated to reduce the carbon flow toward by-products (acetate, alanine, lactate, etc.). These strategies resulted in dramatically decreased by-products and significantly increased L-valine in engineered host overexpressing L-valine production genes (Table 1). 2-ketoisovalerate is the last intermediate of L-valine synthesis, which is also the precursor for L-leucine and D-pantothenate biosynthesis. The enzymes involved in the pathway are KHT and IPMS respectively. To further increase the precursor supply through rational engineering and circumvent the unwanted byproducts, deactivating ketopantoate hydroxymethyltransferase (KHT) and isopropyl malate synthase (IPMS) catalyze the first reaction from 2-ketoisovalerate to D-pantothenate and L-leucine respectively, has been significantly augmented the production of L-valine [45, 46]. Decreasing 2-ketobutyrate availability was suggested also as a practical strategy for efficient pyruvate. Consequently, this can lead to the improvement of L-valine production and preventing the accumulation of L-isoleucine [47]. For instance, AceE is one of the key enzymes encoding pyruvate dehydrogenase complex. Blocking the flow of pyruvate to the TCA pathway leads to pyruvate accumulation and thus more flow to L-valine. Deactivating threonine dehydratase in *aceE*-deficient mutant resulted in the accumulation of L-valine (about 3.5 g L^−1^) compared to *C. glutamicum* ATCC 14067∆*aceE* (about 0.3 g L^−1^) [48]. In addition, applying the similar strategy in *panB* and *panB avtA-*deficient mutant *C. glutamicum* respectively, improved L-valine production [45, 49, 50]. Throughout the construction of L-valine producer strains, the genetic tools rely on complete gene deletion rather than allowing the attenuation of gene expression. To further achieve the optimal production of L-valine, employing the genetic tools including small regulatory RNAs and clustered regulatory interspaced short palindromic repeats interference-nuclease-deactivated Cas9 (CRISPRi-dCas9) is of an urgent need for accurately increasing the precursor pools. Recently, the CRISPR system has implemented in *B. subtilis* to manipulate various key genes to reduce L-valine feedback inhibition and enhance L-valine biosynthesis pathways, resulting in L-valine production up to 4.6 g L^−1^ which is almost 14-fold higher than that obtained with the wild-type strain [16]. Lowering anaplerotic and tricarboxylic acid cycle (TCA) flux through deactivation of pyruvate carboxylase (PCx) and citrate synthase (CS), is thought to lead to the accumulation of pyruvate. By using the CRISPR interference, the expression level of PCx and CS were significantly reduced up to 98 and 83% respectively [51]. Furthermore, CRISPRi is a powerful technology for repressing multiple genes simultaneously in the microbial cells, thus providing a quick and efficient method for reprogramming metabolic pathways [52].

## Cofactor engineering and energy molecules

To further increase precursor supplies toward the pathway of interest, understanding the interplay between cofactor levels/fluxes and metabolic fluxes inside the host is of great importance for efficiency production. Considering a cofactor-dependent production system in microorganisms, two reducing equivalents in form of NAD(P)H are required to synthesize one mole of L-valine from two moles of pyruvate [3]. One mole of NADPH drives the AHAIR enzymatic reduction and another involves in the glutamate dehydrogenase (GDH) reaction which provides the amino group for the final transamination reaction. Although the pentose phosphate pathway (PPP) has higher bioenergetics in terms of ATP and reducing equivalent regeneration, all moles of NADPH generated in PPP are totally consumed during L-valine synthesis and meanwhile conversion of NADH generated via glycolysis to NADPH is being difficult under aerobic conditions. Based on these considerations, cofactor imbalance is a suspected rate-limiting factor in L-valine biosynthesis [53, 54]. The phosphoglucose isomerase (PGI, encoded by *pgi*) catalyzes the production of fructose-6-phosphate from glucose-6-phosphate. To enhance the metabolism of the PPP, Blombach and colleagues demonstrated that deleting the *pgi* gene in *aceE*/*pqo*-deficient *C. glutamicum* significantly increased intracellular NADPH concentration, thereby improved the yield of L-valine up to 0.76 mol mol^−1^ [53]. Bartek and colleagues also applied the similar strategy to improve L-valine yield from 0.49 to 0.67 mol/mol in *C. glutamicum* [54]. Another study demonstrated an adequate alternative to improve NADPH availability in the similar mutant of *C. glutamicum* by overexpressing *E. coli* membrane-bound transhydrogenase PntAB which catalyzes the reversible conversion of NADH to NADPH. This has led to a highly significant increase in L-valine yield up to 0.92 mol mol^−1^ [55]. In *E.coli*, deactivating phosphofructokinase in pyruvate and malate dehydrogenases deficient-mutant increased the metabolite flux through the PPP and enhanced the availability of NADPH [20]. ATP, the major intracellular energy source generated mostly through the TCA cycle coupled with the electron transfer system, is not directly utilized in L-valine biosynthesis under aerobic conditions. Usually, in the process of aerobic fermentation, the energy in the microbial cells is converted into ATP, which maintains the normal metabolism of cells and promotes the growth of the cells. However, in some cases, anaerobic conditions are more favorable for the accumulation of products. Under anaerobic conditions, NADH accumulation and insufficient NADPH may inhibit the production of L-valine. To solve this problem, the Hexose Monophophate Pathway (HMP) can be strengthened and the supply of NADPH can be increased. PpGpp is a regulatory signal molecule in response to environmental changes. *RelA* gene is conducive to the accumulation of ppGpp. When the L-valine synthesis pathway is enhanced, the energy molecule ATP is required. Recently, Denina and colleagues demonstrated that L-valine biosynthesis in *C. glutamicum* was related to *relA* gene that have a positive pact to increase ATP-coupled and decrease ATP-uncoupled respiration [47]. It has also demonstrated that ATP generated by the phosphotransacetylase- acetate kinase pathway plays an important role in L-valine production and cell growth [21]. On the contrary, under anaerobic conditions, ATP is not a constraint to L-valine biosynthesis thus it is not required for enzymatic conversion from pyruvate to L-valine. In recognition of this, Hasegawa and colleagues demonstrated that metabolically engineered *C. glutamicum* strains efficiently produce L-valine under anoxic conditions by converting the coenzyme requirement of AHAIR function from NADPH to NADH, removing feedback inhibition using AHAS variant (G156E), and eliminating byproduct formation for precursor enrichment. The engineered strain produced 172.2 g L^−1^ L-valine with a yield of 0.63 mol mol^−1^ of glucose [56, 57]. Given that a rate-limiting oxygen transfer and carbon loss in form of CO_2_ to generate sufficient reducing equivalent impeded the productivity, applying anaerobic bioprocess suggested a new avenue to the efficient production of L-valine and other amino acids. There is also a two-stage fermentation strategy to produce L-valine (Table 1). After aerobic culture for a period of time, the cells are transferred to a confined space. Under oxygen-limited conditions, in order to improve the regeneration efficiency of cofactors, NADPH is converted to NADH through gene mutations, so that NADH in the EMP pathway can be directly used for the synthesis of L-valine, while introducing foreign genes, replacing the branched-chain amino acid transaminase with the leucine dehydrogenase of *B. subtilis*, changing the dependence on cofactors in the process of L-valine synthesis [56]. Besides, microaerobic environments have been shown to improve the productivity of organisms. Ekaterina A. Savrasova et al. used *E. coli* for L-valine production under microaerobic conditions, this study introduced leucine dehydrogenase Bcd in *B. subtilis*, which altered the NADPH-dependent metabolic pathway, resulting in a final L-valine yield of 9.1 g L^−1^ [58].

## Relieving negative regulatory circuits

The feedback inhibition and transcriptional attenuation control caused by an accumulation of the target product is a common problem encountered in biosynthesis pathways. In this context, the L-valine biosynthesis is then subjected to the negative regulatory circuits which often make it difficult to achieve the optimum production. AHAS is composed of a large subunit (encoded by *ilvB*) and a small subunit (encoded by *ilvN*) which constitute the catalytic domain and the regulatory domain of the enzyme, respectively. Any one of the three branched chain amino acids can bind to the site on the regulatory domain and inhibit the activity of AHAS [23]. Heterologous introduction of feedback-resistant acetolactate acid synthase from *B. Subtilis* increased the production of L-valine [59]. On another hand, the leucine-responsible regulator protein (Lrp) encoded by *lrp* gene, regulates the expression of the AHAS III isoenzyme positively and the AHAS II isoenzyme negatively in *E. coli* [20, 44]. These problems can be solved by mutating this key enzyme to be resistant to feedback inhibition and deregulation of attenuation control. By using site-directed mutagenesis, the feedback inhibition on AHAS was removed to maximize the metabolic flux toward the L-valine biosynthesis. For example; chromosomal expression of AHAS I variant (G41A; C50T) and displacing the attenuator leader region with *tac*-promoter in *E. coli* Val mutant improved the efficiency of 2-ketoisovalerate [20]. Moreover, applying two variants of AHAS (G20D, V21D and M22F) and (P176S, D426E and L576W) conferred L-valine and L-leucine/valine resistance respectively [21, 60]. Removing feedback inhibition in *C. glutamicum* via the chromosomal expression of AHAS variant (G20D, I21D and I22F) has also conferred BCAAs resistance and increased L-valine titer from about 1 g L^−1^ to 15 g L^−1^ in *ilvA* and *panB* deficient mutant [45]. The co-overexpression of L-valine production genes and AHAS variant (S41V and A91V) that conferred BCAAs resistance, resulted in a 9.51 g L^−1^ higher titer and 0.13 g L^−1^ h^−1^ higher L-valine productivity in *C. glutamicum* ATCC 14067 [61]. Hasegawa and colleagues also aimed to relieve the feedback inhibition of L-valine in *C. glutamicum* by developing an active AHAS variant (G156E) (Table1) [57]. In *Saccharomyces cerevisiae*, Takpho and colleagues demonstrated that introducing variant of AHAS (N86A, G89D and N104A) in the host leads to the reduction of the valine-binding affinity thus resulted in approximately 4-fold higher intracellular L-valine contents compared to the wild type [19].

## Balancing gene expression levels

Since the development of DNA recombinant technology along with the use of model organisms, the employment of multiple-copy plasmid-base expression system has substantially improved our ability to design microbial cell factories with a desired phenotype [34]. AHAS is the initiation enzyme of three branched chain amino acids and a key enzyme in the synthesis pathway of L-valine. A limiting step in the production of branched-chain amino acids from metabolically engineered microorganisms can be their excretion from the cell and the export of L-valine is mediated by the exporters BrnFE and YgaZH. Therefore, Overexpression of key enzymes (AHAS, BrnEF, YgaZH) in the rate limiting step is essential for the high yield of L-valine during metabolic engineering [23, 26]. However, fine tuning gene expression approach are capable of achieving high production while minimizing metabolic burden and inefficient carbon utilization, leading to the growth retardation. Considering the enzyme’s function analogies during L-valine and L-isoleucine biosynthesis, balancing the expression of the genes and understanding the ability of the key enzyme to bind to specific intermediates may be the major motivator to maximize the production and maintain the optimal cell growth [32, 62]. Recently, Liu and colleague demonstrated that the 138th residue of *IlvB* in AHAS played an important role for binding the main intermediate pyruvate, thereby overexpression of AHAS variant (V404A, *IlvB*) in YTW-104 mutant increased L-valine production at 25.93 g L^−1^ [63]. In addition, employing the best RBS combinations and promoter engineering are suggested to fine-tune the expression of key biosynthesis genes for L-valine production. Ptrc is a strong promoter that promotes gene expression and BrnFE is responsible for the export of BCCA in *C. glutamicum*. The introduction of Ptrc-controlled *brnFE* in *E. coli* roughly doubled the production of L-valine (32 g L^−1^) [59].

## Transporter engineering

To further enhance the transportability of L-valine, transporter engineering have emerged. In *C. glutamicum*, the BCAAs are transported through a two-component system consisting of BrnF and BrnE encoded by *brnF* and *brnE* respectively. The export of BCAAs and L-methionine is then mediated by BrnFE exporter which is regulated by the global regulator, Lrp (Fig. 1a,b) [64, 65]. In *E. coli*, Lrp is also known to repress the expression of the *livJ* gene, encoding the branched-chain amino acid transporter [20]. Recently, the BCAAs-transporter engineering approach has been implemented to minimize the intracellular concentration of the target product, thus avoiding the feedback inhibition and growth retardation, and ultimately maximizing the production of a target product. For example, in a study by Park and colleagues, the *ygaZH* gene in *E. coli* was found to have a similar function as *brnFE* in *C. glutamicum* through homology research. The overexpression of *ygaZH* and *lrp* further resulted in a 7.61 g L^−1^ L-valine which is 113% higher when compared to the control strain [20]. Other researchers also used the abovementioned strategy in engineered *E. coli* to improve L-valine production (Table 1) [21, 22]. In addition, overexpressing *lrp* and *brnFE* genes in *C. glutamicum* ATCC13869 resulted in increased L-valine production by 16 and 2.7-fold respectively, thus causing the enhanced transcription level of the key genes in L-valine biosynthesis [66]. These studies suggested L-valine transporter engineering is strongly required.

## Co-culture

Recently, L-valine overproduction strains are typically used in strategies based on monoculture. However, the abovementioned discussions indicated that construction and optimization of the L-valine biosynthesis pathway in a single host is still a challenge because of different intrinsic limitations [23, 26]. For instance, many genes in competing pathways are knocked out for enhanced carbon flux, thereby triggering a disproportion of the resources of the host cells. As such, a modular co-culture approach is suggested to overcome the metabolic burdens and helped improve the production level in a cost-effective manner [9]. Generally, the approach based on co-culture is to modularize metabolic pathways and assign pathway modules to more than one microbial strain for the optimal functioning of the complete pathway. In this context, the concept of synthetic microbial consortia is becoming increasingly attractive and the prime benefit of this approach is that the engineered strains shared the overall metabolic burdens which go beyond the metabolic capacity limit of one single strain [9, 67]. Recently, Sgobba and colleagues designed a synthetic *E. coli*-*C. glutamicum* for L-lysine production. Furthermore, commensalism and mutualism-based synthetic consortium for L-lysine production from sucrose and starch respectively were developed [68]. Given the driving force behind the urgent need for microbial conversion of lignocellulosic raw materials, intensive efforts have been made to design synthetic microbial consortia based on lignocellulose sugar for value added chemicals production [69]. More importantly, two user-friendly model hosts of the same species can be engineered to form an integrated system for improved pyruvate intermediate as the main precursor in L-valine biosynthesis. By taking advantage of the co-culture-based approach, enabling co-utilization of mixed sugars through design and characterization of microbial consortia-based L-valine production is suggested to have its impact on industrial application.

## Approaches based on system metabolic engineering

Although the recent progress in rational engineering strategies offers the viable option to enhance the production of L-valine, the key challenge of applying the abovementioned approach is that engineering the microorganisms without considering the complex metabolic and regulatory networks at the system level can lead to the suboptimal production of the target product. Therefore, system metabolic engineering, which integrates system biology, synthetic biology and evolutionary engineering can overcome this challenge [34, 44]. Many tools and strategies are available for implementing systems metabolic engineering; some of these have been applied to L-valine biosynthesis in microbial factories. By applying the in silico-guided multiple knockout approach, the triple knockout of *aceF*, *mdh*, and *pfkA* has potential increased L-valine production in *E. coli* mutant. Consequently, the *E. coli* VAMF strain (Table 1), engineered based on a in silico-prediction target, was able to produce 7.5 g L^−1^ L-valine with a high yield of 0.378 g g^−1^ mol of glucose [20]. In addition to the potential of in silico genome-scale analysis, ^13^C-metabolic flux analysis (^13^C-MFA) can provide a holistic view on metabolism and regulation that is useful for developing L-valine producer strains. For instance, ^13^C-MFA of L-valine producer strain demonstrates that the flux split ratio between EMP and PPP to the demand of NADPH is important for cell growth, maintenance and L-valine production [55]. Hence, a systematic approach based on in silico flux response analysis was applied to identify the optimum substrate feeding condition for increased L-valine production [21].

As discussed, microbial factories possess a plethora of regulatory mechanisms that tightly control the flux through their metabolic network. This mechanism has typically counteracted metabolic engineering efforts to rewire the metabolism with a view to overproduction. Therefore, system metabolic engineering to guided adaptive laboratory evolution (ALE) has become available to optimize microbial genome and finding creative solutions to fitness-limiting obstacles [70]. For example, applying metabolic engineering to guided evolution (MGE), PEP and pyruvate carboxylase-deficient *C. glutamicum* strain was evolved to grow on glucose. The MGE-generated strain showed the mutation in isocitrate dehydrogenase (ICD) which activated the glyoxylate shunt replenishing oxaloacetate required for growth. Furthermore, overexpressing the L-valine biosynthesis gene in evolved strain resulted in 8.9 g L^−1^ L-valine with a yield of 0.22 g g^−1^ mol of glucose [25]. In addition, Omics-based approaches have been applied to acquire valuable transcriptome, proteome and metabolome data, which are being used to develop a strain with higher L-valine production. For example, the genes that are involved in PPP and TCA pathways, and the key genes for biosynthesis of L-valine were found to be upregulated in L-valine higher yield strain, *C.* glutamicum-VWB1 [37]. Ma and colleagues demonstrated that the upregulation of gene encoding aminotransferase might benefit the synthesis of L-valine and cause the significant decrease of byproducts in L-valine-producing mutant *C. glutamicum* XV (Table 1) [71].

Recently, the high-throughput fluorescence activating cell-sorting (HF-FACS) strategy for monitoring and screening single bacterial cells that show a desirable phenotype, has been applied in strain development for amino acids production, including L-valine. A dynamic biosensor regulator system based on Lrp for detection of BCAAs and methionine was developed in *C. glutamicum*. The Lrp upregulated reporter protein expression from the *brnFE* promoter when bound by branched-chain amino acids [72]. Guided by ALE, Mahr and colleagues combined the above sensor system with HF-FACS for screening better mutant of pyruvate dehydrogenase complex-deficient *C. glutamicum* which exhibited a 25% increase in L-valine titer. The detected mutation in a urease accessory protein gene (UreD) and a global protein (GlxR) were responsible for the production phenotype. Furthermore, introducing the mutated sequence of ureD (urease accessory protein) in non-evolved strain resulted in significantly increased L-valine production up to 100% [73]. Based on these examples, hidden constraints on microbial cells lead to undesirable physiological changes. Therefore, a combination of 13C-MF with transcriptomic/proteomics and novel genome-scale models are needed for a comprehensive understanding of cell responses to metabolic burdens at different cellular levels and to design a robust cell with high chances of success. Microbial mutation breeding methods have been widely used in the fermentation industry. Traditional strategies to increase the yield of wild-type strains include random mutagenesis through nitrosoguanidine (NTG) and ultraviolet treatment (UV). In these conventional mutation methods, attention is paid to the health and safety of operators and the efficiency of mutation. Recently, a novel atmospheric and room temperature plasma mutagenesis tool (ARTP) has been developed. ARTP has several advantageous features, such as lower controllable gas temperature, abundant chemically reactive species, rapid mutation, safety and higher operational flexibility. The plasma from the ARTP system damages cell walls, membranes, DNA, and proteins, causing genetic damage in microorganisms. Therefore, ARTP may be a powerful mutagenesis tool providing an effective method to obtain mutants with desirable phenotypes [74]. In addition, rational design combined with biosensor-driven laboratory evolution methods can increase the ability to produce amino acids. A biosensor was constructed based on LRP-type transcriptional regulatory factors and temperature-sensitive replication technology. After atmospheric and room temperature plasma (ARTP) mutagenesis, fluorescence activated cell sorting (FACS) was used to increase the L-valerate of *C. glutamicum*. The yield of the mutant strain L-valine finally obtained was 3.20 g L^−1^, which was 21.47% higher than the titer of the original strain [75].

## Conclusions and perspectives

The branched-chain amino acid, L-valine has wide applications and its market capacity requirement constantly increases, but the production level of this essential biochemical is still far from satisfactory to meet the industrial demands. In this perspective, we describe several strategies that comprise system metabolic engineering for producing L-valine. These strategies encompass carbon source and nutrient utilization, flux distribution at branch points, cofactors and energy requirements, regulatory engineering, product export, gene manipulation, system metabolic engineering, fermentation conditions and characteristics. As engineering of microbial cells for L-valine production is applied more widely, we expect these strategies can be effective in addressing the above challenges.

Although, the use of renewable carbon source, sugar in fermentation with aid of microbial factories offers many advantages as can be seen from the relevant discussion above, several challenges need to be overcome for efficient production. Methanol is a promising raw material for the production of fuels and chemicals. Therefore, people have devoted a great deal of efforts to the design of microorganisms that utilize methanol as an unnatural methyl nutrition platform. The main industrial *C. glutamicum*, after reasonable design and experimental engineering, can be used as a methanol-dependent synthesis of methyl nutrition organisms. The cell growth of methanol-dependent strains depends on the co-utilization of methanol and xylose, most notably methanol is an essential carbon source [76]. The use of abundant and cheap carbon sources can effectively reduce production costs and improve economic feasibility. Acetate is a promising carbon source for realizing cost-effective microbial processes. Recently, some researchers have designed an *E. coli* strain to produce itaconic acid from acetate. In order to increase the yield, the acetic acid assimilation pathway and the glyoxylic acid shunt pathway are amplified through the overexpression and deregulation of pathway genes. After 88 h of fermentation, acetic acid quickly assimilated, and the resulting strain WCIAG4 produced 3.57 g L^−1^ itaconic acid (16.1% of the theoretical maximum yield). These efforts support that acetate may become a potential raw material for engineered *E. coli* production biochemistry [77]. At present,the average selling price of L-valine is $5.4 per kg and the current maximum yield of valine is about 172.2 g L^−1^ L-valine with a yield of 0.63 mol mol^−1^ of glucose for 24 h [59]. The raw materials such as glucose, formic acid and acetic acid are $0.772 per kg, $0.386 per kg and $0.463 per kg respectively. The raw materials such as formic acid and acetic acid obtained from industrial waste are even lower. Therefore, the formate and acetate converted from the reduction of carbon dioxide emissions has great potential and can be used as a sustainable raw material for the biological production of biofuels and biochemical substances. However, due to the toxicity of formate or lack of metabolic pathways, its use for the growth and chemical production of microbial species is limited. The formate assimilation pathway in *E. coli* has been constructed and applied to adaptive laboratory evolution to improve the use of formate as a carbon source under sugar-free conditions [78]. Optimizing the metabolic flow of engineering precursor is frequently complicated between target product and byproduct, gene expression and complex enzyme regulation. Although metabolic problems can be handled by regulating key enzymes, more often than not, the complexity of the branched-chain amino acids synthesis pathway requires more precise regulation. Gene regulation based on CRISPRi has emerged as a powerful tool in synthetic circuits. CRISPRi will have enormous potential as a primary means of technology to reconstruct gene regulatory networks. With the advancement of novel strategies and tools in system metabolic engineering, it is possible to achieve optimal production of L-valine on an industrial scale. The avenues that promise the major impact on L-valine production are: First, stain development by biosensor-mediated adaptive laboratory evolution and high-throughput screening (using biosensors specifically developed for L-valine) [73]. Second, applying the omics strategies, including transcriptomes, proteomes and metabolomes, the physiological information of L-valine producer strain will possibly add insight to hidden constraints. Third, ^13^C-metabolic flux analysis combined with a genome-scale metabolic model (GSM) can lead to the development of optimal process through systematically identifying genetic targets for overexpressions, downregulation and deletions [79]. To this end, all these strategies will provide engineering with insights for improving microbial production for L-valine in the future. Nonetheless, one of the major challenges of metabolic engineering is to transfer the CO_2_ fixation capacity of autotrophic organisms to heterotrophic organisms (such as *E. coli*). The knowledge of more than 100 metabolic pathways of autotrophic *E. coli* can be used, which have been optimized for the production of fuels and chemicals, and are produced through CO_2_. Gleizer et al. in their 2019 contributions type of *E. coli* that can use CO_2_ as the sole carbon source is designed. The author used metabolic engineering technology to construct *E. coli* with disrupted EMP and PPP. The bacteria can fix CO_2_ through the Calvin cycle and use formate as an electronic resource. Then within 350 days, the strain was optimized for CO_2_ fixation through adaptive evolution, thereby generating mutations that regulate gene expression and pathway regulation. Formic acid is introduced into the growth medium. As a method to generate reducing power, formate dehydrogenase is proven to be a key additive to increase the biomass in carbon dioxide from 35 to 100% [43]. All those feedstocks have more possibility to use in microbial production for L-valine.

Industrial production of L-valine requires system-wide engineering, while considering culture conditions in fermentation. It can be summarized as several strategies. Back to the high-yield L-valine, iterative utilization and different orders make more sense. As an increasing body of knowledge on microbial species and metabolic systems which can be implemented in producing L-valine, we expect adopting these strategies can address more challenges, and meanwhile inspirit L-valine development to meet the needs of society.

## Data Availability

All data generated or analysed during this study are included in this published article.

## Abbreviations

CAGR: Compound annual growth rate
BCAAs: Branched-chain amino acids
*C. glutamicum*: *Corynebacterium* *glutamicum*
*E. coli*: *Escherichia* *coli*
*B. subtilis*: *Bacillus* *subtilis*
AHAS: Acetohydroxyacid synthetase
AHAIR: Acetohydroxyacid isomeroreductase
DHAD: Dihydroxyacid dehydratase
TA: Branched-chain amino acid transaminase
FAD: Flavin adenine dinucleotide
PTS: Phosphotransferase system
PEP: Phosphoenolpyruvate
EMP: Embden–Meyerhof–Parnas
PDHC: Pyruvate dehydrogenase complex
KHT: Ketopantoate hydroxymethyltransferase
IPMS: Isopropyl malate synthase
HMP: Hexose monophophate pathway
TCA: Tricarboxylic acid cycle
PCx: Pyruvate carboxylase
CS: Citrate synthase
GDH: Glutamate dehydrogenase
PPP: Pentose phosphate pathway
Lrp: Leucine-responsible regulator protein
ALE: Adaptive laboratory evolution
MGE: Metabolic engineering to guided evolution
ICD: Isocitrate dehydrogenase
HF-FACS: High-throughput fluorescence activating cell-sorting
UreD: Urease accessory protein gene
GlxR: A global protein
NTG: Nitrosoguanidine
UV: Ultraviolet treatment
ARTP: Atmospheric and room temperature plasma
OH: Hydroxyl
FACS: Fluorescence activated cell sorting
GSM: Genome-scale metabolic model
